# New Initiatives at the *WAO Journal*

**DOI:** 10.1097/WOX.0b013e3181a0e5ac

**Published:** 2009-04-15

**Authors:** Lanny J Rosenwasser

## 

Since the January 2009 posting, there have been changes at the *World Allergy Organization Journal *that are worth noting. Prof. Johannes Ring, who led the Journal as Editor-in-Chief in the first critical year of the *WAO Journal*, has moved to the position of Executive Editor. In this position, he will oversee issues related to Editorials that are published in the *WAO Journal*. As Editor-in-Chief, Prof. Ring skillfully handled the transition from the prior *Allergy and Clinical Immunology International Journal *to the new online-only *WAO Journal*. Prof. Ring's effort placed the Journal on the right track to achieve an advantageous position in the pursuit of goals that have been set out for it by the World Allergy Organization (WAO).

Editor-in-Chief responsibilities now reside with Dr. Lanny J. Rosenwasser. In addition to the possibility of easier and immediate worldwide access, the other major benefit of an online journal is the accelerated production process, allowing monthly postings of accepted manuscripts. I am working with the editorial team not only to further streamline the review process for submitted manuscripts but also to continue to reduce production time so that quality manuscripts are made available to readers as quickly as possible.

The overall quality of *WAO Journal *has been excellent and there are a number of new initiatives I have planned for this year to maintain and extend this excellence. To supplement the flow of unsolicited submissions, we will be publishing reviews and papers under three separate content banners. We intend to publish a review series entitled "Clinical Reviews in Allergy and Immunology" with specific clinical relevance and practical measures that are critical for the practice of allergy anywhere in the world. The papers within this series will examine issues of drug and aspirin sensitivity, drug desensitization, natural history of allergic diseases, asthma, atopic dermatitis, and environmental and socio-economic concerns that impact the practice and delivery of care for allergic diseases and that have so far not received the attention they deserve. This series will also provide a vehicle to highlight newer approaches for the evaluation of disease and outcomes concerning treatments and interventions, and keep abreast with evolving developments in informatics, telemedicine, and web-based learning. Focusing on the practical and the new, this series will aim to become indispensable to the clinical practitioner.

A second series of papers will be on "Basic and Clinical Translational Science in Allergy and Immunology." These papers will address pathogenic mechanisms in allergic diseases and examine new, cutting-edge information that extends our understanding of pathogenesis and disease function. Technical advances (including evaluation of systems biology, nanotechnology, and other new approaches to the science of allergy and immunology) and the application of new paradigms and technologies influencing our understanding of the pathogenesis of allergic disease will also be detailed within this series. In keeping with its aim of capturing and discussing the latest advances, this series will have papers on the new roles of cytokines, innate immune system reactions, tissue remodeling, immunopharmacology, pharmacogenomics, and so forth. 

Complementing the practical and the latest will be a more personal series, "Notes of Allergy Watchers," published within the editorial portion of the Journal. This series will invite individuals from our field to share their varied experiences and insights by submitting viewpoints and recollections concerning the science and practice of allergy and immunology. These submissions will be encouraged to include individual personal recollections and interpretation of important issues. The "Notes of Allergy Watchers" will provide not only a look backward but also observations and speculations concerning the science of our discipline and projections for potential changes within our field in the future.

Not every posting will have a selection from each of these three banners, but it is hoped that these will be ongoing series of great interest for the practice and science of allergy and immunology and WAO members all over the world. The worldwide platform of the *WAO Journal *offers a great opportunity for exchange of information and ideas among different regions of the world. But it is worth remembering that we are all dedicated to the care of patients with allergic diseases, and the *WAO Journal *is dedicated to promoting any activity that enhances our capabilities in this area and extends new knowledge and information.

